# Controlled and Targeted Drug Delivery Using Smart Nanovectors

**DOI:** 10.53941/ijddp.0201010

**Published:** 2023-03-20

**Authors:** Abou Bakr M. Salama, Yasmin Y. Salem, Tamer M. A. Mohamed

**Affiliations:** 1 Institute of Molecular Cardiology, Department of Medicine, University of Louisville, KY 40208, U.S.A.; 2 Faculty of Medicine, Zagazig University, Zagazig 44519, Egypt.; 3 Envirome Institute, Centre for Cardiometabolic Sciences, Department of Medicine, University of Louisville, KY 40208, U.S.A.; 4 Department of Bioengineering, Speed School of Engineering, University of Louisville, KY 40208, U.S.A.; 5 Department of Pharmacology and Toxicology, University of Louisville, KY 40208, U.S.A.; 6 Institute of Cardiovascular Sciences, University of Manchester M13 9PL, U.K.

## Abstract

The conventional drug delivery systems have several limitations, such as the high frequency of administration, several off-target effects, and the need for tissue specificity. Recently, smart drug shuttles have emerged, and the nano applications provided a new opportunity for advancing the drug delivery system to become tissue targeted and decrease the frequency of administration. The recent development of nanovectors as drug carriers has gone through several steps of evolution that ended with the development of logic-embedded nanovectors. Here, we summarize the different types of nanovectors and their applications in various clinical situations, and finally, we spot the light on the future of this area of research.

## Graphical Abstract

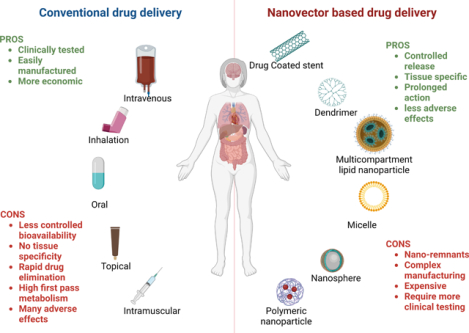

## 1. Overview and Perspectives

### 1.1. Introduction

Over the past few decades, drug development has witnessed revolutionary steps regarding pharmacokinetics, pharmacodynamics, and pharmaceutical aspects. Yet, the conventional drug delivery methods, either inhalational, parenteral, enteral, or subdermal, had too many limitations, including less bioavailability, loss of tissue specificity, and rapid drug degradation or elimination [1,2]. This urge for more potent drugs with fewer side effects, better bioavailability, and higher tissue selectivity has provoked efforts to generate innovative new drug delivery methods. One of the unique cutting-edge technologies under detailed investigation is the use of nanoparticles for drug delivery [3]. Recent developments in nanotechnology have guaranteed the development of different nanoscale particles from different chemical compounds or elements with unique, novel chemical and physical properties [4 – 9]. Interestingly, recent efforts focused on developing nanoparticle-based vectors, also named “smart nanovectors,” as they can overcome previously used conventional methods. Most drugs under investigation as cargo for nano-delivery methods are cancer drugs [10–14]. The long list includes, from the simple nanoliposomes and micelles to the more complex multistage delivery systems and nano-electro-mechanics [15,16]. This review highlights the major findings in this hot area and aims to draw attention to the possible future applications beyond the oncological uses of smart nanoparticles.

### 1.2. Shortcomings of the Conventional Drug Delivery Methods

Drug delivery is the process that entails the administration of a particular pharmaceutical compound to gain a therapeutic benefit [17]. The process of drug development, though not young, is highly sophisticated and should bear in mind many aspects, including the drug’s physiochemical properties, drug effects, body effects and interactions, and patient satisfaction and compliance. The conventional routes for drug administration include but are not limited to oral, rectal, subcutaneous, intramuscular, and intravascular routes [18,19]. The oral route is convenient, noninvasive, and encourages compliance. Still, too many shortcomings are encountered, including the degradation by the gastrointestinal tract (GIT) enzymes, the first pass metabolism, food interactions, irregular absorption, and the low solubility and permeability of some drugs administered [20,21]. For example, oral intake is not suitable for protein drugs such as insulin, a drug used by 26 % of the diabetic population, which is approximately 25.8 million people in the USA [22].

The intravenous administration of drugs achieves 100% bioavailability and bypasses the first-pass metabolism with a rapid response to the drug. Furthermore, it is still an invasive method that needs a trained person to deliver the drug under sterile conditions with a high possibility of toxicity. All the conventional methods also need more tissue specificity, meaning that they are distributed to all tissues with high too many adverse effects encountered and low drug concentration at the target tissue, a problem that necessitates increasing the administrated dose with more side effects encountered [23].

### 1.3. Types of Nanovectors

A wide range of nano-delivery systems (nanovectors) with different physical, chemical, and geometrical properties are under development and investigation, which can be grouped under three main subsequent generations [24–26]. The first generation is mainly elementary vectors that bypass the gastrointestinal enzymes and target tissues by enhanced permeation and retention effect (EPR) with prolonged circulation time and reduced immunogenicity with some chemical modifications such as adding the natural polymer polyethylene glycol (PEG). This category includes liposomes, micelles, dendrimers, and polymer nanoparticles [27–30]. Further developments to enhance tissue targeting include surface antibodies, aptamers, or oligonucleotides that bind to unique, overexpressing receptors on the target tissue. Other efforts include co-delivering several drugs and/or diagnostic materials with simultaneous, sequential, triggered, or controlled release. These advances have led to the development of the second generation of nanovectors [31–35]. The third generation of the nanovectors is called Logic Embedded Vectors (LEVs), or smart nanovectors, as stated by Serda et al., “therapeutic, multi-component constructs specifically engineered to avoid biological barriers, in which the functions of biorecognition, cytotoxicity, and bio-barrier avoidance are decoupled, yet act in efficacious, operational harmony” [24,36,37]. This means that multiple nano-components collaborate to generate a time sequence of events. This generation includes multistage delivery systems, nano-cells, and silicon-based delivery systems, or what is known as mesoporous silicon [34,35,38,39].

### 1.4. Tissue Targeting

Many strategies have been developed to increase the affinity of certain pharmaceutical compounds loaded on a nanovector toward particular tissue. These pharmaceutical compounds have either diagnostic, therapeutic, or theranostic applications. In targeted therapeutic agents, the efficacy of the target tissue is increased by reducing toxicity to the other organs and tissues [40]. On the other hand, imaging compounds targeting the tissues leads to better delineation of the target [41]. The concept of tissue targeting depends upon unique expression or overexpression of specific ligands on the cell surface; for example, vascular endothelial growth factor receptor (VEGF) and integrins expression on the tumor cell surface have been used as a target for nanovectors through coating the surface of the nanovectors with peptides, thioaptamers, carbohydrates or antibodies. Khemtong *et al*. used the peptide of Arg-Gly-Asp-D-Phe-Lys (cRGD) to actively target integrins on tumors [42]. Yoo et al. developed another targeting strategy using folic acid on the surface of the nano shuttles to bind to the overexpressed folic acid receptors on the tumor cell surface [43]. Carbohydrates are well-studied for active tissue targeting. For example, the hepatocellular carcinoma cells are overexpressing the asialoglycoprotein receptor (ASGPR) that can be targeted by either galactose or lactose, as demonstrated by Cho et al. [44]. Monoclonal antibodies are an efficient tool for tissue targeting with high specificity.

The FDA has approved many drugs depending on this idea, including trastuzumab, an anti-Human epidermal growth factor receptor-2 (anti-HER2) used in HER2-positive breast cancer [45]. Aptamers are oligonucleotides that can exhibit 3D folding, which has a high binding affinity binding to cell surface proteins, making them an excellent targeting molecules. The thio-modification of these oligos increases their stability and resistance to nucleases and facilitates their intracellular transport by reducing the negative charges. The systematic evolution of ligands can synthesize aptamers by exponential enrichment (SELEX) technology [46] and thio-modified either enzymatically or chemically later on. Thioaptamers-coated nanovectors are currently under development [47–49].

## 2. Novel Therapeutic Applications of the Smart Nanovectors

### 2.1. Nanovectors and Anticancer Cargos

The list of efforts to develop a selective anticancer drug delivery is growing daily, with many of the above mentioned technologies being applied [50]. Two independent investigators developed a pH-responsive nanovector for doxorubicin (DOX) delivery to tumor tissues. In one study, the drug was encapsulated in polysebacic anhydride (PSA) as a nanovector which was mixed with pH-sensitive poly L-histidine (PLH), while the polyethylene glycol (PEG) was used to reduce the phagocytosis of the designed particles. In the other study, amphiphilic four-arm star-polymers-poly-(e-caprolactone)-b-poly-(2-(diethylamino) ethylmethacrylate) was used. These studies showed a pH-dependent drug release profile with the promising intracellular release of the cargo [51,52].

Another strategy used is the thermo-sensitive nanovectors such as the recently developed thermosensitive biotinylated hydroxypropyl cellulose-based polymer micelles (HPC-PEG-Chol-biotin). The prepared micelles delivered paclitaxel (PTX) to cancer cells. Bagheri *et al*. studied the PTX-carrying micelles in vitro and showed a temperature-dependent release profile of the drug with strong adsorption to the cancer cells [53]. In another study, paclitaxel co-loaded with carboplatin on lipid-polymer hybrid nanoparticles was used for cervical cancer treatment [54]. Finally, thermostable RNA-based nanoparticles were utilized for PTX treatment of breast cancer [55].

Temozolomide (TMZ), a prodrug for a DNA alkylating agent used to treat glial tumors, was delivered using a smart targeting nanoconjugate. In this study, Polymalic acid was used as a platform with PEG as an anti-phagocytic molecule, monoclonal antibody to the transferrin receptors, and tri-leucine for pH-dependent endolysosomal release of the TMZ. Patil et al. proved that TMZ polymer nanoparticles entered the tumor cells effectively with a specific release of the cargo in the endolysosomes, meaning that these particles can be used effectively in tumor targeting [56].

Many nanovectors’ shuttled drugs were recently approved by the FDA for clinical use, and several other strategies are still under clinical trials[57]. For example, PTX loaded on albumin nanoparticles (Abraxane) was approved for treating breast cancer, non-small cell lung cancer, and pancreatic cancer [58–60]. In addition, Doxil loaded on liposomes was approved for treating Kaposi sarcoma, ovarian cancer, and multiple myeloma [61].

### 2.2. Nanovectors for Brain Diseases

The delivery of drugs to the brain faces a particular obstacle known as the blood-brain barrier (BBB). The conventional routes for brain drug delivery include disruption of the BBB, intraventricular/intrathecal injection, and intranasal administration. However, nanovector-based delivery has provided a new opportunity for brain-targeted drug delivery [62]. For example, the hexapeptide dalargin (Tyr-D-Ala- Gly- Phe-Leu-Arg), which has an opioid-like activity, was the first drug to be delivered to the brain using nanocarriers [63]. Most recently, a Transferrin receptor-1 targeted nanovectors were used to treat brain tumors [64].

### 2. 3. Nanovectors for Cardiovascular Therapeutics

Cardiovascular diseases (CVD) are increasing in developed countries, with billions of dollars in costs and millions of people affected. Several recent efforts to develop new therapeutic strategies for CVD with higher efficacy and fewer adverse effects. Hence the nanovectors carry great promise; there is a growing effort to create nanovectors-based CVD therapeutics [64]. Several examples include targeting selectins, especially vascular cell adhesion molecule-1 (VCAM-1) and intracellular adhesion molecule-1 (ICAM-1), or fibrins to deliver the therapeutics to the exposed plaques in the atherosclerotic vessels [65–67]. Tölli *et al*. studied the possibility of using porous silicon biomaterials as nanovectors to deliver drugs to cardiac tissues, especially after myocardial infarction (MI). They used rat models to deliver the nanoparticles before and after induction of MI and studied the effect on the hematological indices, blood flow, cardiac functions, inflammatory markers, and post-MI fibrosis with promising results that encouraged using such particles as shuttles [68]. Clinically, polymers are widely used as drug carriers in CVD in the drug-eluting stents (DES) to treat coronary artery stenosis and atheromatous plaques. Boston scientific corporation introduced Taxus^®^ DES, a polymer of styrene-block-isobutylene-block-styrene (SIBS) coated with an antiproliferative agent to prevent the recurrence of coronary stenosis, and the list of DESs in clinical utility is growing every day [69–71].

### 2.4. Nanovectors and Lung Diseases

Pulmonary artery hypertension (PAH) is among the most challenging diseases, with a few therapeutic modalities available. Iloprost, a well-known drug for PAH, can be administered by inhalation to minimize its side effects, but it is easily degraded, which dictates frequent dosing. 5(6)-carboxyfluorescein (CF) nanoparticles, a new class of biocompatible, fast degrading, branched polyesters, were examined as a convenient method for sustained delivery of iloprost [72,73].

### 2.5. Infectious Diseases and Nanovectors

Most antibiotics, antifungal, and anti-parasitic agents have several side effects, especially nephrotoxicity. One of the studies by Kotwani and others addressed the delivery of amphotericin B through a liposomal vector [74]. This formula is safe in patients with renal impairment and resistance to conventional antifungal drugs with a lower dose of the drug required in comparison with the other formulas. Also, a nanovector was studied for drug delivery in visceral leishmaniasis with the great success achieved [74–76]. FDA approved AmBisome as a liposomal system for amphotericin B delivery as a treatment of fungal infections [77].

### 2.6. Nanovectors for Controlled Drug Release

Together with tissue-specific targeting, nanoparticles can be used for other purposes, including bypassing the low pH of the stomach and the first-pass metabolism, specific drug release in the distal gut, and controlled drug release and pulsatile release. For example, β-Cyclodextrin conjugated polyethyleneimine (PEI-CD) was used as a carrier to deliver indomethacin to the colon, acting as gastro-off /intestinal-on formulas [78]. Also, unique colon delivery methods have been developed for treating inflammatory bowel diseases using Sulfasalazine, ipsalazide, and olsalazine [79–86].

Several studies have addressed using chitosan/alginate nanovectors for oral insulin administration. The oral delivery of insulin will be of great advantage over subcutaneous injections. At the stomach, alginate forms dense networks preventing insulin degradation by the proteolytic enzymes, while in the intestine, the chitosan adheres to the mucosa facilitating insulin release and absorption [87–90].

Chiang and his colleagues developed PLGA-based nanocarriers capable of pulsatile cargo release on exposure to magnetic fields. Doxorubicin (DOX) was in an aqueous core surrounded by iron oxide magnetic nanoparticles in the polymer shell. The drug release is said to be switched on and off by exposure to external magnetic stimuli. The same mechanism can be used for the drugs requiring initial boluses to reach the therapeutic concentrations and then steady dosing to maintain its level in tissues or blood [91].

## 3. Future Perspective

The clinical need for smart nanovectors in various applications is increasing. Many of the recently used conventional drugs are currently under consideration again for nanovectors delivery. The drugs that should be highly prioritized are to treat deadly diseases such as cancer and CVD to avoid adverse effects. The field of smart nanovectors research is growing every day, and great success is being achieved. Yet, the number of commercially available formulations is still low compared to the ongoing research. This reflects a failure of many of the proposed models to fulfill the clinical practice needs for several reasons. Issues like biosafety of the proposed nanoparticles, excretion/metabolism of the nano-remnants, and commercial feasibility of shifting to nanoparticle-based therapies should be considered when designing the next generations of nanovectors.

## List of abbreviations:

ASGPR: asialoglycoprotein
BBB: blood brain barrier
CF: carboxyfluorescein
CVD: cardiovascular diseases
DES: drug eluting stents
DOX: doxorubicin
EPR: enhanced permeation and retention
FDA: food and drug administration
GIT: gastrointestinal tract
HER: Human epidermal growth factor receptor
ICAM: Intercellular Adhesion Molecule
LEVs: Logic Embedded Vectors
MI: myocardial infarction
PAH: Pulmonary artery hypertension
PEG: poly ethylene glycol
PEI-CD: Cyclodextrin conjugated polyethyleneimine
PLGA: poly lactic co glycolic acid
PLH: poly L-histidine
PSA: polysebacic anhydride
PTX: paclitaxel
SELEX: systematic evolution of ligands by exponential enrichment
SIBS: styrene-block-isobutylene-block-styrene
TMZ: Temozolomide
VCAM: vascular cell adhesion molecule
VEGF: vascular endothelial growth factor
